# Calculated identification of mutator-derived lncRNA signatures of genomic instability to predict the clinical outcome of muscle-invasive bladder cancer

**DOI:** 10.1186/s12935-021-02185-3

**Published:** 2021-09-08

**Authors:** Yingchun Liang, Fangdie Ye, Zhang Cheng, Yuxi Ou, Lujia Zou, Yun Hu, Jimeng Hu, Haowen Jiang

**Affiliations:** 1 Departments of Urology, Huashan Hospital, Fudan University, No. 12 WuLuMuQi Middle Road, Shanghai, 200040 China; 2 Fudan Institute of Urology, Huashan Hospital, Fudan University, Shanghai, China; 3 National Clinical Research Center for Aging and Medicine, Fudan University, Shanghai, China

**Keywords:** Genomic instability, Mutator phenotype, Long non-coding RNAs, Muscle-invasive bladder cancer

## Abstract

**Background:**

Muscle-invasive bladder cancer (MIBC) is one of the most important type of bladder cancer, with a high morbidity and mortality rate. Studies have found that long non-coding RNA (lncRNA) plays a key role in maintaining genomic instability. However, Identification of lncRNAs related to genomic instability (GIlncRNAs) and their clinical significance in cancers have not been extensively studied yet.

**Methods:**

Here, we downloaded the lncRNA expression profiles, somatic mutation profiles and clinical related data in MIBC patients from The Cancer Genome Atlas (TCGA) database. A lncRNA computational framework was used to find differentially expressed GIlncRNAs. Multivariate Cox regression analysis was used to construct a genomic instability-related lncRNA signature (GIlncSig). Univariate and multivariate Cox analyses were used to assess the independent prognostic for the GIlncSig and other key clinical factors.

**Results:**

We found 43 differentially expressed GIlncRNAs and constructed the GIlncSig with 6 GIlncRNAs in the training cohort. The patients were divided into two risk groups. The overall survival of patients in the high-risk group was lower than that in the low-risk group (P < 0.001), which were further verified in the testing cohort and the entire TCGA cohort. Univariate and multivariate Cox regression showed that the GIlncSig was an independent prognostic factor. In addition, the GIlncSig correlated with the genomic mutation rate of MIBC, indicating its potential as a measure of the degree of genomic instability. The GIlncSig was able to divide FGFR3 wild- and mutant-type patients into two risk groups, and effectively enhanced the prediction effect.

**Conclusion:**

Our study introduced an important reference for further research on the role of GIlncRNAs, and provided prognostic indicators and potential biological therapy targets for MIBC.

**Supplementary Information:**

The online version contains supplementary material available at 10.1186/s12935-021-02185-3.

## Background

Bladder cancer is the ninth most common tumor in the world. The prevalence rate accounts for 7% of all cancers, and the mortality rate accounts for 3% of all cancers [1]. Muscle-invasive bladder cancer (MIBC) accounts for approximately 20% of newly diagnosed bladder cancer cases. Approximately 15%–20% of non-muscular invasive bladder cancers progress to MIBC. The 5-year overall survival (OS) rate of MIBC patients is about 60% to 70% [2, 3]. Males have a higher incidence, but female patients may already be in a more advanced stage at the time of diagnosis, resulting in a lower survival rate [4]. Advanced MIBC may spread to nearby lymph nodes or other organs, and even to distant organs, such as lungs and liver [5]. Although the current treatment methods include surgery, radiotherapy, chemotherapy, and immunotherapy, the recurrence rate of patients is relatively high and the survival rate of advanced patients is low [6–8]. Invasion and metastasis of MIBC are important factors for recurrence and poor prognosis [9, 10]. Due to the complex molecular mechanism and clinical heterogeneity within the tumor, the therapeutic effect is different. Therefore, a new biomarker is urgently needed to more accurately assess the clinical prognosis of MIBC.

Studies have reported that genomic instability (GIN) and gene mutations are one of the hallmarks of cancer [11, 12]. GIN can be observed in a variety of malignant tumors and pre-cancerous lesions [13]. The accumulation of GIN is closely related to tumor progression and survival, and is an important prognostic factor [14, 15]. At the same time, the drug resistance of tumor is also related to GIN [16, 17]. For example, chromosomal mutant of the epidermal growth factor receptor gene can make glioblastoma cells resistant to epidermal growth factor receptor inhibitors [17]. At the same time, studies have found that excessive GIN or certain gene mutation can also hinder cell growth and make cancer cells sensitive to drugs. This may be due to excessive genotoxicity and protein toxicity [18, 19]. For example, cells with BRCA gene defects are sensitive to ionizing radiation [20, 21]. These opposite effects, as well as the selective killing cancer cells of GIN, indicate that GIN is both a challenge and a potential opportunity for all kinds of cancer treatment [22, 23].

Although we do not yet fully understand the molecular basis of GIN, abnormal transcription and post-transcriptional regulation are closely related to GIN [24]. Molecular markers have the potential to be able to quantitatively detect GIN. For example, Habermann et al. tested the gene expression profiles of 48 breast cancer patients and designed a GIN signature consisting of 12 genes [25]. Wang et al. constructed miRNA regulatory networks that is associated with DNA damage responses, and identified 10-miRNA signature related to outcome of ovarian cancer and GIN [26–28].

Long non-coding RNA (lncRNA) is a type of RNA that has no protein-coding ability and is usually more than 200 nt [29]. Studies have shown that it can regulate gene expression during or after transcription [30]. Many lncRNAs have specific expression profiles in specific cancers, and the abnormal expression of lncRNAs may play a key role in the occurrence, proliferation, progression or metastasis of tumor [31]. More and more evidences show that lncRNA plays a vital role in maintaining GIN [32]. For example, Mendell et al. showed that DNA damage can activate specific lncRNAs, interact with proteins involved in DNA repair and replication, and then maintain genomic stability [33]. Betts et al. confirmed that two lncRNAs, CUPID1 and CUPID2, can regulate the expression of genes related to DNA repair [34]. LncRNA DDSR1 can maintain genome stability by regulating the expression of DNA damage genes or binding to DNA damage proteins [35, 36]. Although existing studies have confirmed that several lncRNAs are related to genome stability, lncRNAs related to genomic instability (GIlncRNAs) and their clinical guidance value in cancer are still largely unexplored.

In this study, we tried to construct a genomic instability-derived lncRNA signature (GIlncSig) combining lncRNA expression profiles and somatic mutation profiles, and to evaluate and verify its prognostic ability and independent prognostic value, so as to guide clinical application of the GIlncSig in MIBC.

## Meterials and methods

### Data collection

The clinical characteristics, RNA-seq expression data and somatic mutation information of MIBC patients were collected from the Cancer Genome Atlas (TCGA) database (https://portal.gdc.cancer.gov/). A total of 399 cases with paired lncRNA and mRNA expression profiles, somatic mutation information and clinicopathological parameters including individual survival status, survival time, T stage, N stage, M stage, age and gender were obtained. All MIBC patients used in this study were randomly divided into two groups, named training cohort and testing cohort respectively. A total of 200 patients in the training cohort were used to establish a prognostic-related risk model. The testing cohort (n = 199) and the entire TCGA cohort (n = 399) were used to verify the prognostic-related risk model. Table 1 shows brief information on clinical and pathological characteristics. We checked the relevant data of FGFR3 mutation patients on the cBioPortal.

**Table 1:** Clinical and pathological characteristics of MIBC patient cohorts in this study

Covariates	Training cohort (n = 200)	Testing cohort (n = 199)	TCGA cohort (n = 399)	P-value
Age, no (%)				
Young (≤ 65)	74 (37%)	85 (42.71%)	159 (39.85%)	0.2876
Old (> 65)	126 (63%)	114 (57.29%)	240 (60.15%)	
Gender, no (%)				
Female	57 (28.5%)	48 (24.12%)	105 (26.32%)	0.3791
Male	143 (71.5%)	151 (72.88%)	294 (73.68%)	
Grade, no (%)				
High Grade	189 (94.5%)	187 (93.97%)	376 (94.24%)	0.8185
Low Grade	9 (4.5%)	11 (5.53%)	20 (5.01%)	
Unknown	2 (1%)	1 (0.5%)	3 (0.75%)	
Stage, no (%)				
Stage II	64 (32%)	62 (31.16%)	126 (31.58%)	0.9413
Stage III–IV	135 (67.5%)	136 (68.34%)	271 (67.92%)	
Unknown	1 (0.5%)	1 (0.5%)	2 (0.5%)	
T, no (%)				
T2	62 (31%)	55 (27.64%)	117 (29.32%)	0.5967
T3–4	123 (61.5%)	126 (63.32%)	249 (62.41%)	
Unknown	15 (7.5%)	18 (9.05%)	33 (8.27%)	
M, no (%)				
M0	89 (44.5%)	103 (51.76%)	192 (48.12%)	0.8268
M1	6 (3%)	5 (2.51%)	11 (2.76%)	
Unknown	105 (52.5%)	91 (45.73%)	196 (49.12%)	
N, no (%)				
N0	120 (60%)	113 (56.78%)	233 (58.4%)	0.872
N1–3	63 (31.5%)	63 (31.66%)	126 (31.58%)	
Unknown	17 (8.5%)	23 (11.56%)	40 (10.03%)	
Fustat, no (%)				
Alive	114 (57%)	109 (54.77%)	223 (55.89%)	0.7286
Dead	86 (43%)	90 (45.23%)	176 (44.11%)	

### Identification of genomic instability-associated lncRNAs

In order to identify lncRNAs related to genomic instability (GIlncRNAs), we used a calculation framework derived from the mutation hypothesis designed by Bao et al. [37]. It is roughly as follows, we calculated the number of somatic mutations in each patient and ranked the patients in descending order of the number of somatic mutations. The bottom 25% of patients were defined as the genomic stable (GS) group, and the top 25% of patients belonged to the genomic unstable (GU) group. The lncRNA expression profile of the two groups was compared using the 'limma' package of R version 4.0.2. Differentially expressed lncRNAs (fold change > 1, false discovery rate (FDR) adjustment P < 0.05) were defined as GIlncRNAs.

### Identification of a genomic instability-derived lncRNA signature

Univariate Cox regression proportional hazard analysis was performed on the expression level of GIlncRNAs to find the lncRNA related to prognosis in MIBC patients. Then these GIlncRNAs were included in the multivariate Cox regression to obtain the correlation coefficient. The GIlncSig was constructed to predict the outcome. The risk score was calculated by the following formula: Risk score =  (-0.291 × Expression of LINC02446) +  (-0.368 × Expression of AC078880.3) +  (0.018 × Expression of LINC01419) +  (0.328 × Expression of AC087392.1) +  (0.197 Expression of LINC02762) +  (0.100 × Expression of CFAP58-DT). The median value of patients in the training cohort was used as a risk cutoff point, patients were divided into high-risk groups and low-risk groups.

### Pathway enrichment analysis

We used the 'limma' package in R to find mRNAs paired with GIlncRNAs, considered the top 10 mRNAs as lncRNA-related partners, and constructed a co-expression network. In order to predict the potential function of lncRNA, we used the 'clusterProfiler' and 'ggplot2' packages in R to perform Kyoto Encyclopedia of Genes and Genomes (KEGG) pathway enrichment analysis on the mRNAs co-expressed with lncRNAs.

### Cell culture and siRNA knockdown

We obtained bladder cancer (T24 and 5637) cell lines from the Type Culture Collection in Chinese Academy of Sciences (Shanghai, China). T24 cells were cultured in Dulbecco’s modified Eagle medium (Gibco, Grand Island, NY, USA) and 5637 cells were cultured in 1640 medium (Gibco), supplemented with 10% foetal bovine serum (FBS, Gibco) in a humidified incubator at 37 °C with 5% CO2. We transfected si-CFAP58-DT, and respective negative controls into cultured T24 and 5637 cells using Lipofectamine 2000 (Invitrogen, Carlsbad, CA, USA) following standard procedure. Following transfection for 48 h, the follow-up experiments were carried out. The sequence for si-CFAP58-DT was as follows: CGCCTCTAATTCTCCAATACATC.

### Cell proliferation and apoptosis assays

We used the Cell Counting Kit 8 (Gibco) assay to assess cell proliferation. We placed 2000 transfected cells per well into 96-well plates in triplicate wells for 5 days and detected cell proliferation at 1, 2, 3, 4, and 5 d after seeding according to the standard manufacturer’s instructions.

We used the Annexin V-APC/7-AAD Apoptosis Kit (MultiSciences; Hangzhou China) to detect apoptosis according to the manufacturer's instructions. In short, after washing in PBS, cells were incubated in 7-AAD staining solutions and APC Annexin-V in the dark at room temperature for 5 min. After incubation, the cells were collected by a C6 flow cytometer (BD, NY, USA) and FlowJo 10.4 software was used for analysis. Apoptotic cells population include 7-AAD- and APC Annexin-V +  (undergoing apoptosis) cells and 7-AAD + and APC Annexin-V +  (end of apoptosis or death) cells.

### Statistical analysis

The 'survfit' and 'survdiff' functions in the 'survival' package of R were used to compare the survival differences between the high- and low-risk groups. The Kaplan–Meier method was used to present the survival rate of each prognostic risk group. The performance of the GIlncSig was evaluated by a time-dependent receiver operating characteristic (ROC) curve. Univariate and multivariate Cox analyses were used to assess the independent prognostic for the GIlncSig and other key clinical factors. All statistical analyses were handled with R version 4.0.2.

## Results

### Identification of genomic instability-related lncRNAs in MIBC patients

**Fig. 1:**
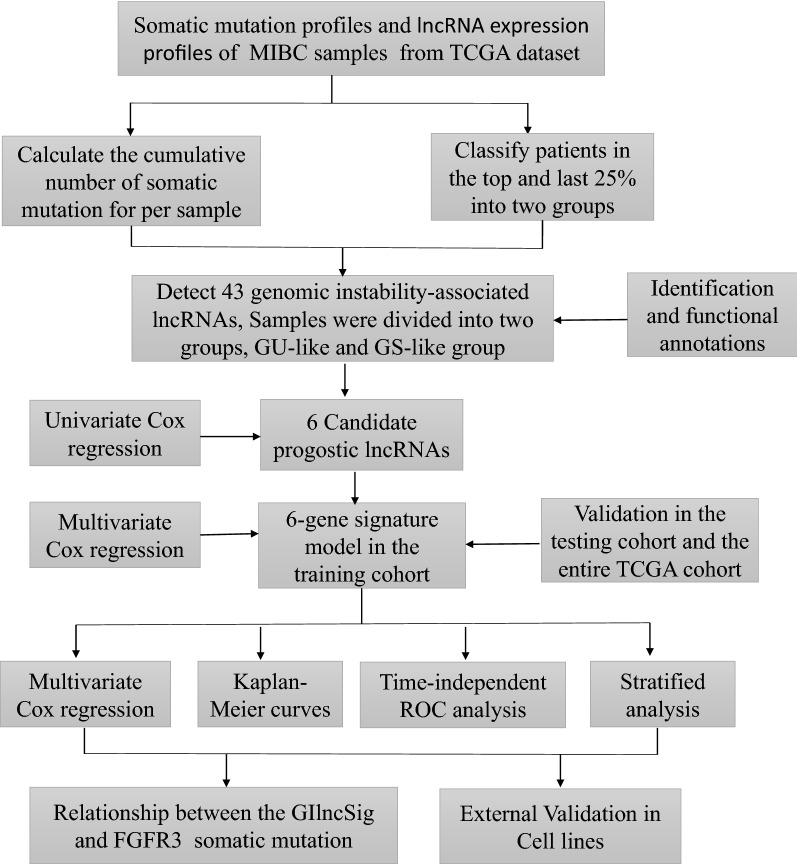
Workflow of this study

**Fig. 2:**
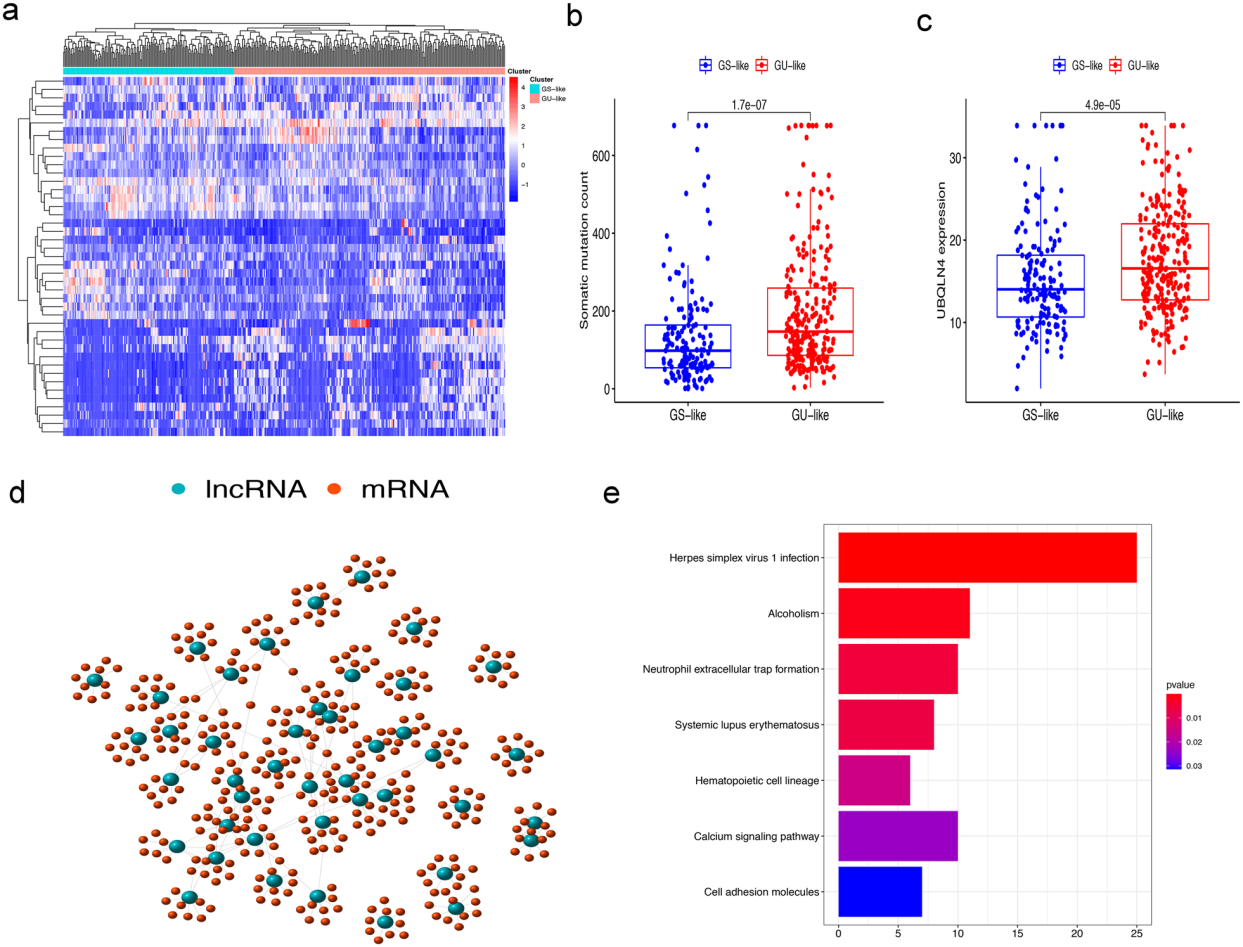
Identification and functional annotation of GIlncRNAs in MIBC patients. **a** Based on the expression patterns of 43 genomic instability-related lncRNAs, unsupervised clustering of 399 MIBC patients. The blue cluster on the left is GS-like group, and the red cluster on the right is GU-like group. **b** Boxplots of somatic mutations in the GS-like group and GU-like group. The accumulation of somatic mutations in the GS-like group is significantly lower than that in the GU-like group. **c** Boxplots of the expression level of UBQLN4 in the GU-like group and GS-like group. The expression level of UBQLN4 in GS-like group was significantly lower than that in GU-like group. **d** Co-expression network of differential lncRNA related to genomic instability and their corresponding mRNAs. The blue circles represent lncRNAs, and the red circles represent mRNAs. **e** KEGG pathway analysis the main signalling pathways for mRNAs related to differential lncRNAs

The Workflow in Fig. 1 is a brief summary of this research. In order to extract lncRNAs related to GIN, we divided the patients into GS-like group and GU-like group according to the cumulative number of somatic mutations in each patient. Then the lncRNA expression profiles between the two groups were compared. There were a total of 43 significantly differentially expressed lncRNAs (Additional file 1: Fig. S1a). All 399 samples were divided into two groups based on the expression levels of 43 differentially expressed lncRNAs (Fig. 2a). The group with low accumulation of somatic mutations was called GS-like group, and the other group was GU-like group. The median value of somatic accumulated mutations in the GS-like group was significantly lower than that in the GU-like group (P < 0.001, Fig. 2b). We then compared the expression levels of the UBQLN4 gene (a driver of GIN) in the GS-like and GU-like group. The expression of UBQLN4 in the GS-like group was significantly lower than that in the GU-like group (P < 0.001, Fig. 2c).

### Enrichment analysis

In order to determine whether these lncRNAs were related to GIN, we performed a functional enrichment analysis of its related genes to predict potential functions. First, the top 10 mRNAs of 43 differentially expressed lncRNAs were obtained, and the lncRNAs-mRNA co-expression network was constructed (Fig. 2d). The KEGG pathway enrichment analysis was performed on the mRNA co-expressed by lncRNA. Seven significantly enriched pathways were found, most of which are related to GIN, including Alcoholism and Systemic lupus erythematosus (Fig. 2e). These results indicated that 43 differentially expressed lncRNAs were related to GIN, and these differentially expressed lncRNAs were defined as candidate GIlncRNAs.

### Development of a GIlncSig for outcome prediction in the training cohort

**Fig. 3:**
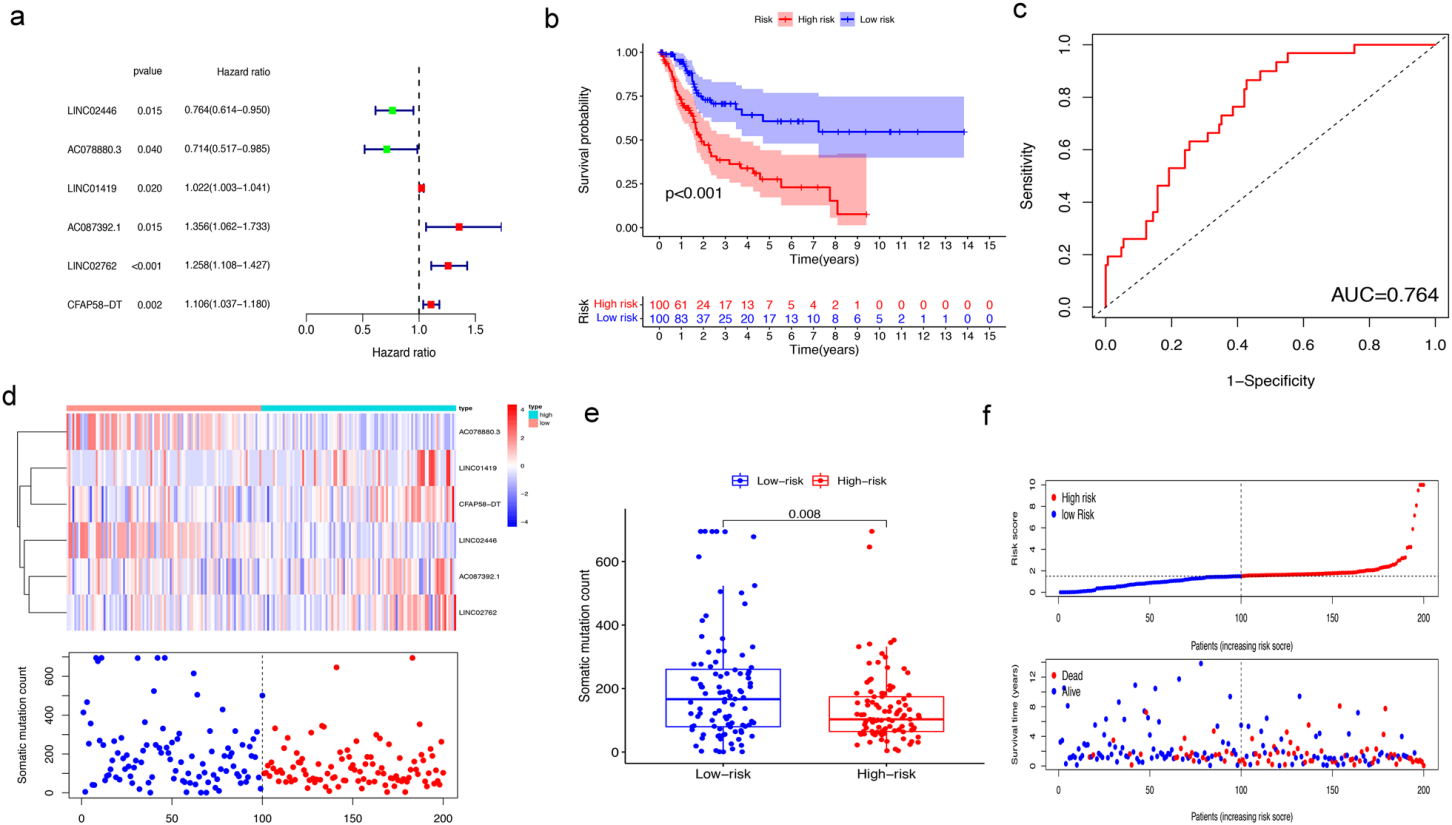
Evaluation of the genomic instability-related lncRNA signature (GIlncSig) in the training cohort. **a** Kaplan–Meier survival curve estimates of overall survival of high-risk and low-risk MIBC patients. **b** Forest plot shows the results of the univariate Cox analysis for the 6 genomic instability-related lncRNAs. **c** ROC curve and AUC based on the training cohort at 5 years. **d** Expression heatmap of six lncRNAs and the distribution of somatic mutation. **e** Boxplots of the distribution of somatic mutations in the high-risk and low-risk groups. **f** Distribution of risk score, survival outcome of patients based on data

To further research the prognostic effects of these GIlncRNAs, we randomly divided 399 MIBC samples into a training cohort (n = 200) and a testing cohort (n = 199). Univariate Cox proportional hazard regression was used to analyze the relationship between the expression levels of 43 GIlncRNAs and OS in the training cohort. It was found that the expression levels of 6 GIlncRNAs were significantly correlated with the prognosis of MIBC patients (P < 0.05, Fig. 3a). Then, multivariate Cox analysis was used to construct the GIlncSig. Table 2 summarized the detailed information of 6 lncRNAs including their Ensembel ID, coefficients and the results of multivariate Cox regression analysis. Among the GIlncSig, the coefficients of lncRNA LINC01419, AC087392.1, LINC02762 and CFAP58-DT were positive, indicating that they may be risk factors, and their high expression was related to a poor prognosis. While lncRNA LINC02446 and AC078880.3 may be protective factors, and their high expression showed a better prognosis. We used the above formula to obtain the risk score of each patient in the training cohort, and then divided these patients into a high-risk group and a low-risk group based on the threshold of the median risk score. Kaplan–Meier analysis indicated that the survival results of patients in the high-risk group were significantly lower than those in the low-risk group (P < 0.001, Fig. 3b). The time-dependent ROC curve analysis of the GIlncSig showed that the area under the curve (AUC) was 0.764 (Fig. 3c). We sorted the patients according to the risk score, and observed the expression level of the GIlncSig, the count of somatic mutations, and the risk score in the patients. For patients with high risk scores, the expression level of risk lncRNAs were up-regulated, while the expression level of protective lncRNAs were down-regulated. Conversely, the patients with low scores showed the opposite result (Fig. 3d). Comparison analysis indicated that there were significant differences in the somatic mutation patterns of patients in the low-risk group and the high-risk group. The number of somatic mutations in the high-risk group was significantly lower than that in the low-risk group (P = 0.008, Fig. 3e). The risk score, survival time and survival status of each patient in the training cohort were shown in Fig. 3f.

**Table 2:** Regression coefficient and P-value of 6 genomic instability-related lncRNAs (GIlncRNAs) based on multivariate Cox regression

ID	coef	HR	95%CI	P-value
LINC02446	-0.291	0.748	0.597–0.937	0.012
AC078880.3	-0.368	0.692	0.497–0.963	0.029
LINC01419	0.018	1.018	0.999–1.037	0.067
AC087392.1	0.328	1.389	1.088–1.772	0.008
LINC02762	0.197	1.218	1.078–1.377	0.002
CFAP58-DT	0.100	1.105	1.035–1.181	0.003

### Validation of the GIlncSig in the testing cohort and the entire TCGA MIBC cohort

**Fig. 4:**
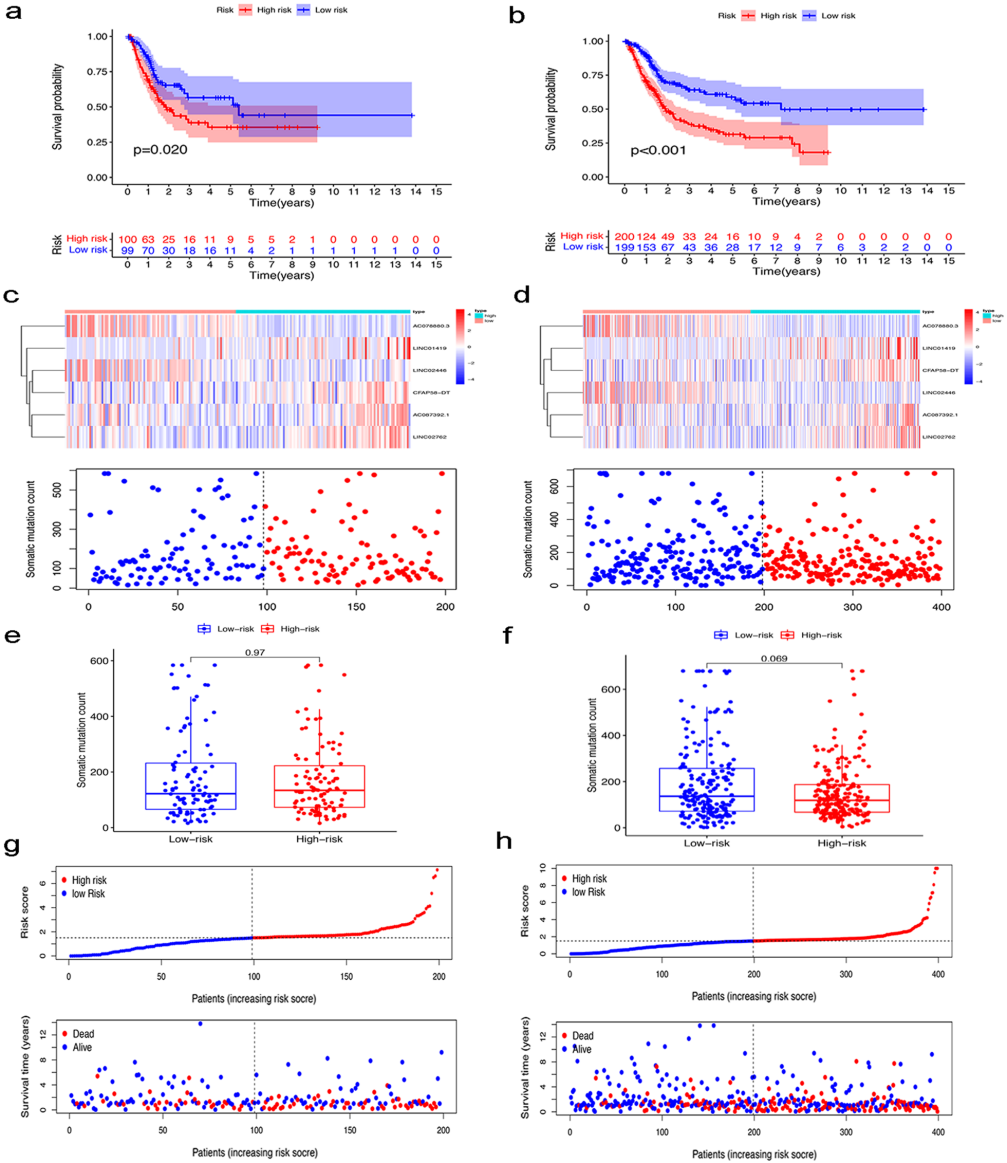
Evaluating the predictive power of the GIlncSig in the testing cohort and entire TCGA cohort. Kaplan–Meier survival curve estimates of overall survival of high-risk and low-risk groups in the testing cohort (**a**) and entire TCGA cohort (**b**). Expression heatmap of six lncRNAs and the distribution of somatic mutation in the testing cohort (**c**) and entire TCGA cohort (**d**). Boxplots of the distribution of somatic mutations in the testing cohort (**e**) and entire TCGA cohort (**f**). Distribution of risk score, survival outcome of patients based on data in the testing cohort (**g**) and entire TCGA cohort (**h**)

In order to examine the predictive ability and stability of the GIlncSig, we verified it in the testing cohort (n = 199) and the entire TCGA MIBC cohort (n = 399). The same GIlncSig and risk thresholds derived from the training cohort was applied to the testing cohort and the entire TCGA MIBC cohort. The 199 patients in the testing cohort were divided into a high-risk group (n = 100) and a low-risk group (n = 99). The overall survival of patients in the high-risk group were significantly lower than those in the low-risk group (P = 0.020, Fig. 4a). The time-dependent ROC curve analysis of the GIlncSig in the testing cohort showed that the AUC was 0.630 (Additional file 1: Fig. S1b). The distribution of somatic mutation counts and the expression level of the GIlncSig in the testing cohort samples were shown in Fig. 4c. The number of somatic mutations in the high-risk group and the low-risk group were similar (P = 0.97, Fig. 4e). The risk score, survival time and survival status of each patient in the testing cohort were shown in Fig. 4g.

The prognostic and prediction performance of the GIlncSig in the entire TCGA MIBC cohort was similar to the previous results. Patients in the entire TCGA MIBC cohort were divided into a high-risk group (n = 200) and a low-risk group (n = 199). The survival time of patients in the high-risk group were significantly lower than those in the low-risk group (P < 0.001, Fig. 4b). The time-dependent ROC curve analysis of the GIlncSig in the entire TCGA MIBC cohort showed that the AUC was 0.686 (Additional file 1: Fig. S1c). The distribution of somatic mutation counts and the expression level of the GIlncSig in the entire TCGA MIBC cohort samples were shown in Fig. 4d. The number of somatic mutations in the high-risk group was lower than that in the low-risk group (P = 0.069, Fig. 4f). The risk score, survival time and survival status of each patient in the entire TCGA MIBC cohort were shown in Fig. 4h.

### Independent prognostic analysis of the GIlncSig and clinical relevance analysis

**Fig. 5:**
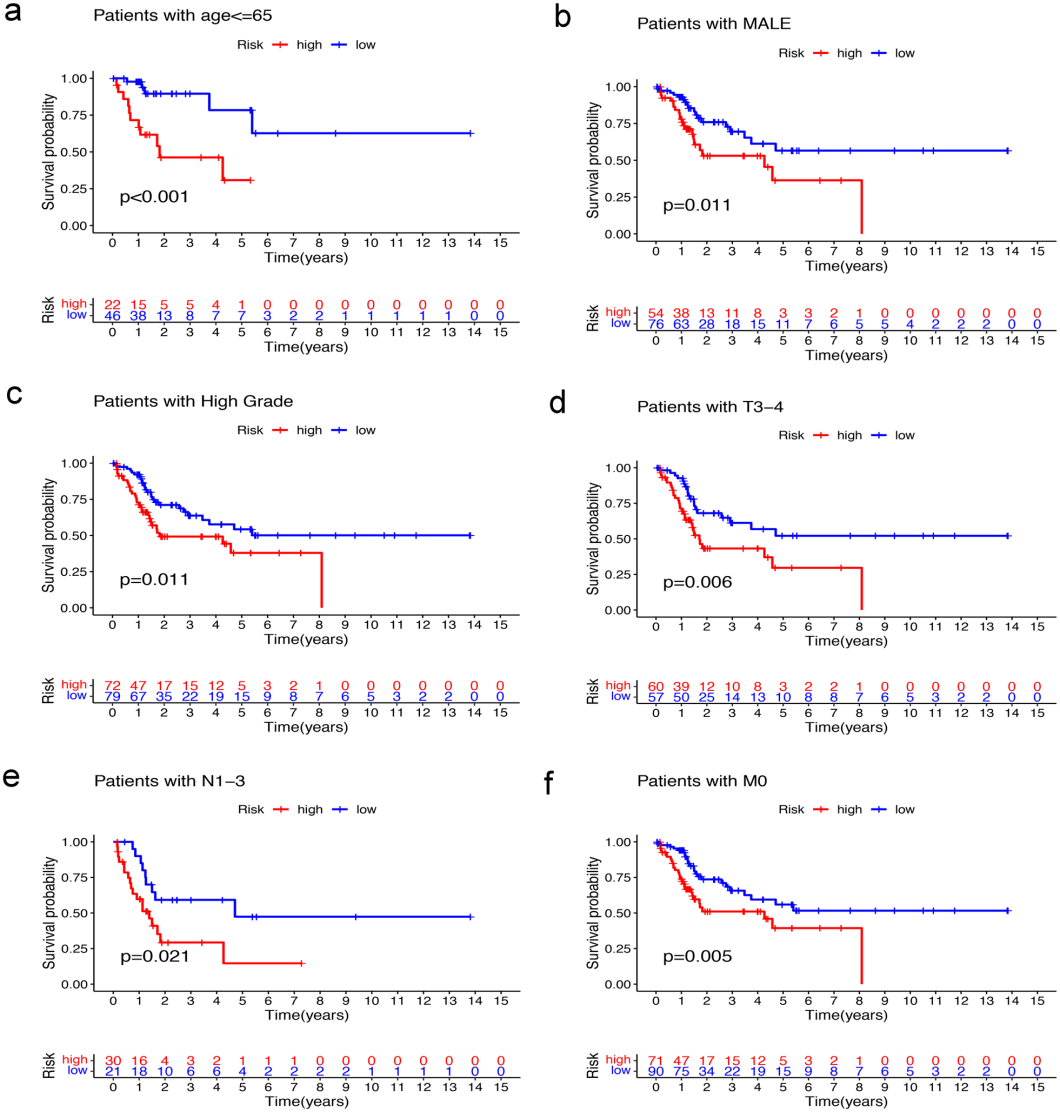
Stratification analyses of the GIlncSig. Kaplan–Meier survival curve estimates of overall survival of high-risk and low-risk groups in patients ≤ 65 years **a**, male patients (**b**), high grade patients (**c**), T3–4 stage patients (**d**), N1–3 patients (**e**), and M0 patients (**f**)

To assess the independent prognostic value of the GIlncSig and its relationship with common clinical variables, multivariate Cox analyses were applied on age, gender, pathological grade, pathological stage, and the GIlncSig risk score model. The results showed that the GIlncSig was significantly related to the OS in each cohort (Table 3). Therefore, it is determined that the GIlncSig was an independent prognostic indicator for MIBC patients. Except for the GIlncSig, other indicators also have important prognostic values. In order to further verify, we carried out a stratified analysis of each index. Briefly, we divided the patients into young patients and elderly patients according to age, with 65 years old as the demarcation point. Similarily, the patients were divided into male and female groups by gender, divided into high-grade and low-grade groups according to pathological classification. Subsequently, the patients were divided into early group (stage T2) and advanced group (stage T3–4), without lymph node metastasis (stage N0) group and lymph node metastasis (stage N1–3) group, without distant metastasis (stage M0) group, and distant metastasis (stage M1) group. Then the GIlncSig was used to divide the patients in each group into high-risk or low-risk groups. Finally, it was found that in the elderly patient group, male group, high-grade group, T3–4 group, N1–3 group and M0 group, the OS rate of the high-risk groups were significantly lower than those of the low-risk groups (P < 0.05; Fig. 5a-f).

**Table 3:** Univariate and Multivariate Cox regression analysis of the GIlncSig in different cohorts

Variables		Univariable model			Multivariable model			
		HR	95%CI	P-value	HR	95%CI		P-value
Training cohort (n = 203)								
Age	(> 65)/ (≤ 65)	2.082	0.788–5.503	0.139				
Gender	Male/female	0.918	0.388–2.174	0.846				
T	(T3–4)/T2	1.884	0.648–5.475	0.244				
M	M1/M0	2.280	0.530–9.813	0.357				
N	(N1–3)/N0	2.743	1.285–5.854	0.009	2.244	1.019–4.940		0.045
Risk score	High/low	1.625	1.314–2.009	** < 0.001**	1.541	1.238–1.918		** < 0.001**
Testing cohort (n = 200)								
Age	(> 65)/ (≤ 65)	1.458	0.697–3.051	0.317				
Gender	Male/female	0.454	0.217–0.948	0.037	0.510	0.241–1.079		0.078
T	(T3–4)/T2	2.673	1.023–6.988	0.045				
M	M1/ M0	2.427	0.574–10.273	0.228				
N	(N1–3)/N0	1.935	0.945–3.959	0.071				
Risk score	High/low	1.980	1.338–2.931	** < 0.001**	1.910	1.266–2.882		**0.002**
TCGA cohort (n = 403)								
Age	(> 65)/(≤ 65)	1.629	0.915–2.901	0.098				
Gender	Male/female	0.635	0.364–1.108	0.109				
T	(T3–4)/T2	2.197	1.078–4.480	0.030				
M	M1/ M0	2.108	0.759–5.856	0.152				
N	(N1–3)/N0	2.222	1.325–3.725	0.002	1.657	0.932–2.944		0.085
Risk score	High/low	1.563	1.344–1.817	** < 0.001**	1.489	1.274–1.739		** < 0.001 **

### Combination of the GIlncSig and FGFR3 mutation status

**Fig. 6:**
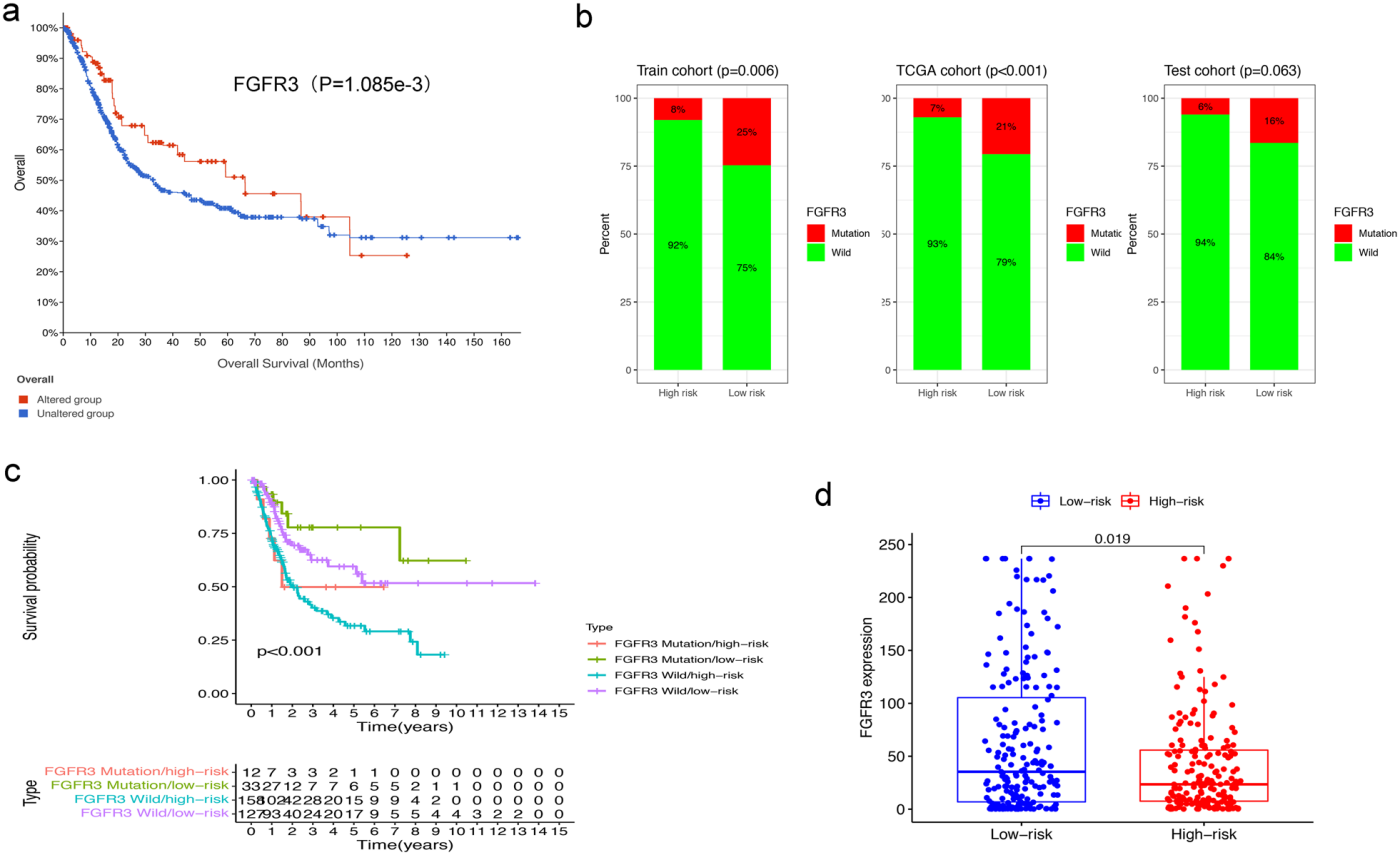
Relationship between the GIlncSig and FGFR3 somatic mutation. **a** Kaplan–Meier survival curve for BCa patients stratified by the FGFR3 mutation. **b** The proportion of FGFR3 mutation in high-risk and low-risk groups in the training cohort, entire TCGA cohort and testing cohort. **c** Kaplan–Meier curve of overall survival is shown for MIBC patients classified according to FGFR3 mutation status and the GIlncSig. **d** FGFR3 expression in patients of low-risk and high-risk groups in the entire TCGA cohort

FGFR3 mutations have an important value in bladder cancer [38]. From cBioPortal, we found that the OS of patients with FGFR3 mutations was better than that of wild-type ones (p < 0.05, Fig. 6a). Further analysis showed that in the training, testing and entire TCGA MIBC cohort, the proportion of FGFR3 mutation patients in the high-risk group was significantly lower than that in the low-risk group (Fig. 6b). The results demonstrated that the GIlncSig was also related to the FGFR3 mutation status and may be a mutation marker of the FGFR3. Therefore, we further tested whether the GIlncSig combined with FGFR3 mutation status could better predict the outcome. We used the GIlncSig and FGFR3 mutation status to divide patients into four risk groups, which were FGFR3 Mutation/high-risk group, FGFR3 Mutation/low-risk group, FGFR3 Wide/high-risk group and FGFR3 Wide/low-risk group. The survival curves of the four groups were significantly different (P < 0.001, Fig. 6c). The FGFR3 Mutation/low-risk group had the best survival results. The results showed that the GIlncSig can effectively distinguish the survival results of FGFR3 Mutation and FGFR3 Wide patients respectively. At the same time, we tested the relationship between FGFR3 mRNA expression and the GIlncSig. It was found that the higher the expression level of FGFR3 mRNA, the lower the risk of patients (P = 0.019, Fig. 6d).

### Validation of gene expression and function

**Fig. 7:**
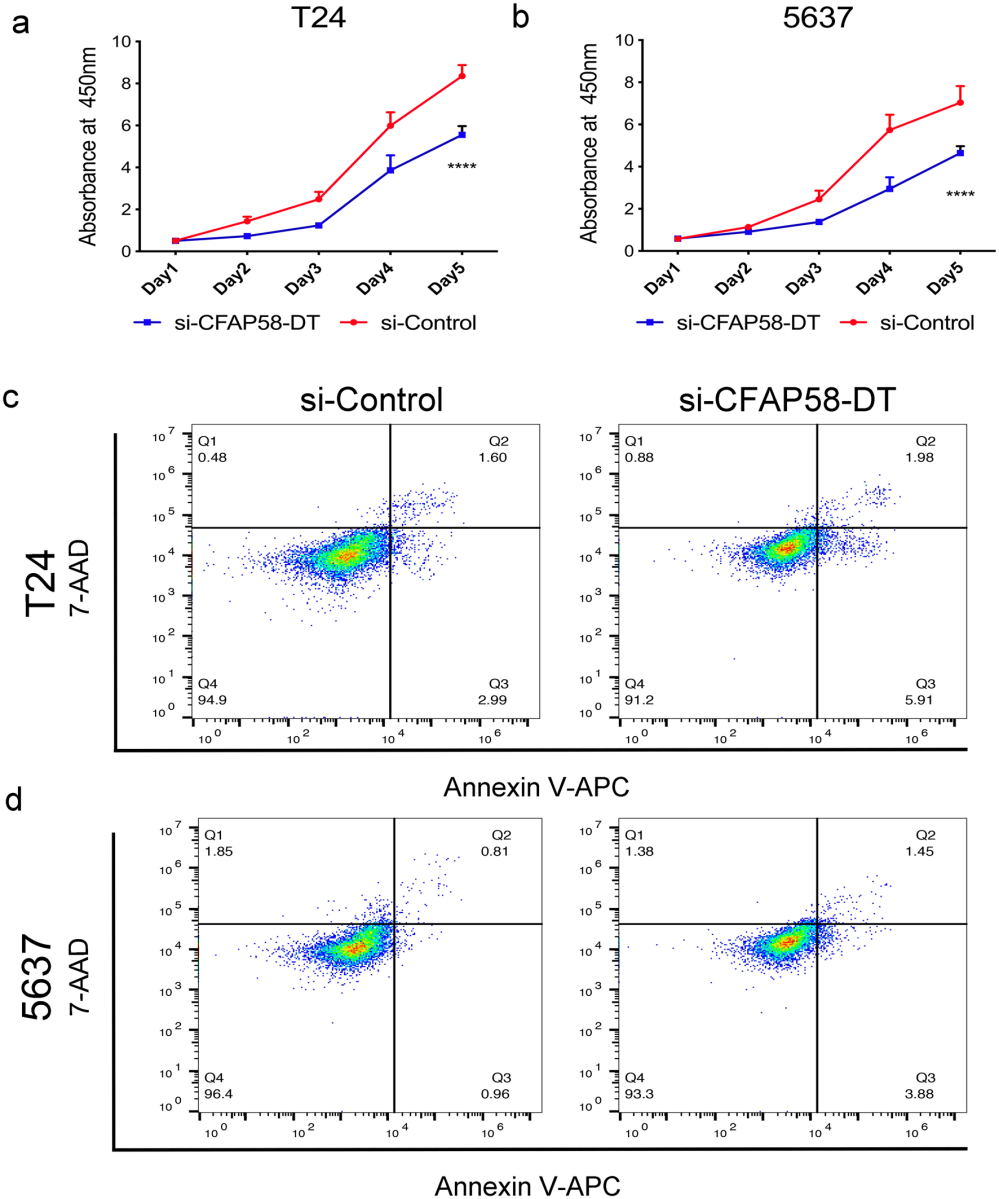
Experimental verification of CFAP58-DT. CCK-8 assay results show the relative proliferation of si-CFAP58-DT- and si-control-transfected T24 (**a**) and 5637 (**b**) cells. Apoptosis rates of si-CFAP58-DT- and si-control-transfected T24 (**c**) and 5637 (**d**) cells were detected by 7-AAD-Annexin V staining and analyzed by flow cytometry

Next, we used the CFAP58-DT gene to validate our model. CCK-8 proliferation assays showed that compared with si-control T24 and 5637 cells, the proliferation of CFAP58-DT-knockdown T24 and 5637 cells was significantly reduced (P < 0.05, Fig. 7a, b). The apoptotic rate of CFAP58-DT-knockdown T24 cells was (7.567 ± 0.397) %, and the apoptotic rate of si-control T24 cells was (4.687 ± 0.097) %, the difference was statistically significant (P < 0.05, Fig. 7c). The apoptotic rate of CFAP58-DT-knockdown 5637 cells was (5.32 ± 0.1) %, and the apoptotic rate of si-control 5637cells was (1.92 ± 0.2) %, the difference was statistically significant (P < 0.05, Fig. 7d).

## Discussion

GIN plays an important role in the occurrence, progression and recurrence of cancer. The pattern and degree of GIN are of great significance to the diagnosis and prognosis of tumors [39, 40]. However, the quantitative measurement of the degree of GIN has always been a challenge. Evidence shows that the abnormal gene transcription and replication and inactivation of DNA repair can lead to GIN [41]. Although a large number of studies have reported that GIN plays an important role in the occurrence and development of tumors, studies have found that excessive GIN may hinder cancer cell growth and reduce the risk of disease [11]. This study found that the GIlncSig was correlated with the genomic mutation rate of MIBC, indicating its potential as a measure of GIN. Moreover, the number of mutations in the low-risk group was higher than that in the high-risk group, which provided an important reference for further judgment of the disease prognosis.

In recent years, lncRNA is an important part of tumor biology. It is closely related to the occurrence and progression of tumors, and can be used as a new type of prognostic marker [42, 43]. For example, LncRNA GClnc1 is associated with the progression of bladder cancer [44]. In addition, lncRNAs are closely related to genome stability. Studies have found that lncRNA NORAD [45] and GUARDIN [46] play an important role in maintaining the stability of the genome. However, there are few studies on the relationship between lncRNA and cancer related to GIN. Therefore, we combined lncRNA expression profiles and somatic mutation profiles, found out GIlncRNAs, and then constructed a GIlncSig to predict the survival prognosis in MIBC. In the training cohort, the GIlncSig divides patients into high- and low-risk groups, which was verified on the testing and entire TCGA MIBC cohorts. This may be used as a promising prognostic indicator of MIBC, and may find specific GIlncRNAs in MIBC, which can provide an important reference for the occurrence, development and prognosis of the disease.

First, we screened 43 GIlncRNAs and performed a pathway enrichment analysis on their co-expressed mRNAs, which were mainly enriched in Alcoholism, Systemic lupus erythematosus. These pathways were related to gene instability [47–50]. The GIlncSig in this study was composed of 6 lncRNAs. LINC01419, AC087392.1, LINC02762 and CFAP58-DT were risk factors and were related to poor prognosis, while LINC02446 and AC078880.3 were protective factors and were related to better prognosis. According to the results of Kaplan–Meier survival analysis, the GIlncSig had a strong prognostic performance and was significantly associated with the prognostic survival of MIBC patients. Using univariate and multivariate Cox regression analysis, we conducted independent prognostic analysis of risk factors such as age, gender, stage and the GIlncSig. It was found that risk score was an independent prognostic factor of MIBC. It indicates that it may be superior to other clinicopathological features and can be used as a better prognostic biomarker. Further stratified analysis showed that in the elderly patient group, male group, high-grade group, T3–4 group, N1–3 group and M0 group, the OS rate of the high-risk groups were significantly lower than those of the low-risk groups. The GIlncSig can predict the survival prognosis of patients in each subgroup more accurately.

Among the 6 lncRNAs in the GIlncSig, AC087392.1, AC078880.3 and LINC02762 have not been reported. LINC01419 can promote the growth and metastasis of hepatocellular carcinoma [51], gastric cancer cells [52] and osteosarcoma cells [53]. Xie et al. found that CFAP58-DT can enhance the innate immune response to viral infection [54], but it has not been reported in tumor treatment. Research by Zhang et al. showed that LINC02446 inhibits the proliferation and metastasis of bladder cancer cells [55]. We further analyzed the effect of CFAP58-DT on bladder cancer cells. The CCK-8 proliferation test showed that compared with si-control T24 and 5637 cells, the proliferation ability of CFAP58-DT-knockdown T24 and 5637 cells was significantly reduced. Flow cytometry found that the apoptosis rate of CFAP58-DT-knockdown T24 and 5637 cells was significantly increased. This finding indicates that CFAP58-DT may play a carcinogenic effect in bladder cancer, but the specific mechanism needs to be further studied. This model not only plays an important role in predicting the prognosis of MIBC, but also provides a new biomarker to guide the clinical treatment of MIBC patients.

We added a correlation analysis between the FGFR3 mutation and risk score to enhance the prognostic performance of our GIlncSig. This is not available in previous studies. A large number of studies have confirmed that FGFR3 mutation is a common genetic activity in bladder cancer and has become a promising molecular target for recurrence, prognosis, and treatment targets in bladder cancer [56, 57]. FGFR3 mutations in bladder cancer may have better OS [38]. Consistent with previous studies, the FGFR3 mutation rate of patients in the high-risk group was significantly lower than that in the low-risk group, suggesting that the GIlncSig may be able to predict FGFR3 mutation status. In addition, we also observed that the GIlncSig can significantly distinguish the different clinical outcomes of FGFR3 wild-type and mutant-type patients and the FGFR3 Mutation/low-risk group has the best survival results. These results indicated that the combination of the GIlncSig may have better prognostic significance than FGFR3 mutation status alone. At the same time, we observed that the higher the expression level of FGFR3 mRNA, the lower the risk of patients, which may be due to the increased transcription of FGFR3 caused by FGFR3 mutation. The specific mechanism needs further study.

Although our research provides an effective method to better evaluate GIN and an important reference for the prognosis of MIBC patients. There are still some limitations. Firstly, our GIlncSig was constructed and verified in the TCGA data cohort, more independent data cohorts and biological experiments are needed to verify its robustness and reproducibility. Secondly, it is necessary to use prospective clinical data to verify its clinical applicability, to ensure its stability and reproducibility, and to explore its potential mechanism of action.

## Conclusions

In conclusion, the GIlncSig was constructed by combining the lncRNA expression profiles, the somatic mutation profiles and clinical information of MIBC, which can be used as an independent prognostic marker for MIBC patients and successfully stratified the risk subgroups of MIBC patients. The combination of the GIlncSig and FGFR3 mutation status can improve the predictive effect of patients. Understanding the underlying mechanism and significance of the GIlncSig in MIBC can provide advice for determining the therapeutic target of MIBC.

## Supplementary Information

**Additional file 1: Fig. S1.** (a) Heatmap of expression of 43 differential lncRNAs. (b) ROC curve and AUC based on the testing cohort at 5 years. (c) ROC curve and AUC based on the entire TCGA MIBC cohort at 5 years.

## Data Availability

All data generated or analyzed were downloaded from TCGA database (https://portal.gdc.cancer.gov/repository?facetTab=cases).

## Abbreviations

MIBC: Muscle-invasive bladder cancer
TCGA: The Cancer Genome Atlas
lncRNA: Long non-coding RNA
GIlncRNA: LncRNAs related to genomic instability
GIlncSig: Genomic instability-related lncRNA signature
FGFR3: Fibroblast growth factor receptor 3
OS: Overall survival
GIN: Genomic instability
GS: Genomic stable
GU: Genomic unstable
KEGG: Kyoto Encyclopedia of Genes and Genomes
ROC: Receiver operating characteristic

## References

[1] Kamat AM, Hahn NM, Efstathiou JA, Lerner SP, Malmstrom PU, Choi W, Guo CC, Lotan Y, Kassouf W (2016). Bladder cancer. Lancet.

[2] Patel VG, Oh WK, Galsky MD (2020). Treatment of muscle-invasive and advanced bladder cancer in 2020. CA Cancer J Clin.

[3] Grainger S, Traver D, Willert K (2018). Wnt Signaling in Hematological Malignancies. Prog Mol Biol Transl Sci.

[4] Dobruch J, Daneshmand S, Fisch M, Lotan Y, Noon AP, Resnick MJ, Shariat SF, Zlotta AR, Boorjian SA (2016). Gender and bladder cancer: a collaborative review of etiology, biology, and outcomes. Eur Urol.

[5] Hanna KS (2017). A review of immune checkpoint inhibitors for the management of locally advanced or metastatic urothelial carcinoma. Pharmacotherapy.

[6] Redelman-Sidi G, Glickman MS, Bochner BH (2014). The mechanism of action of BCG therapy for bladder cancer–a current perspective. Nat Rev Urol.

[7] Williams SB, Shan Y, Ray-Zack MD, Hudgins HK, Jazzar U, Tyler DS, Freedland SJ, Swanson TA, Baillargeon JG, Hu JC (2019). Comparison of costs of radical cystectomy vs trimodal therapy for patients with localized muscle-invasive bladder cancer. JAMA Surg.

[8] Fonteyne V, Ost P, Bellmunt J, Droz JP, Mongiat-Artus P, Inman B, Paillaud E, Saad F, Ploussard G (2018). Curative treatment for muscle invasive bladder cancer in elderly patients: a systematic review. Eur Urol.

[9] Lightfoot AJ, Breyer BN, Rosevear HM, Erickson BA, Konety BR, O'Donnell MA (2014). Multi-institutional analysis of sequential intravesical gemcitabine and mitomycin C chemotherapy for non-muscle invasive bladder cancer. Urol Oncol.

[10] Choueiri TK, Raghavan D (2008). Chemotherapy for muscle-invasive bladder cancer treated with definitive radiotherapy: persisting uncertainties. Nat Clin Pract Oncol.

[11] Zhang W, Mao JH, Zhu W, Jain AK, Liu K, Brown JB, Karpen GH (2016). Centromere and kinetochore gene misexpression predicts cancer patient survival and response to radiotherapy and chemotherapy. Nat Commun.

[12] Negrini S, Gorgoulis VG, Halazonetis TD (2010). Genomic instability–an evolving hallmark of cancer. Nat Rev Mol Cell Biol.

[13] Pihan GA, Wallace J, Zhou Y, Doxsey SJ (2003). Centrosome abnormalities and chromosome instability occur together in pre-invasive carcinomas. Cancer Res.

[14] Ottini L, Falchetti M, Lupi R, Rizzolo P, Agnese V, Colucci G, Bazan V, Russo A (2006). Patterns of genomic instability in gastric cancer: clinical implications and perspectives. Ann Oncol.

[15] Suzuki K, Ohnami S, Tanabe C, Sasaki H, Yasuda J, Katai H, Yoshimura K, Terada M, Perucho M, Yoshida T (2003). The genomic damage estimated by arbitrarily primed PCR DNA fingerprinting is useful for the prognosis of gastric cancer. Gastroenterology.

[16] Lee AJ, Endesfelder D, Rowan AJ, Walther A, Birkbak NJ, Futreal PA, Downward J, Szallasi Z, Tomlinson IP, Howell M (2011). Chromosomal instability confers intrinsic multidrug resistance. Cancer Res.

[17] Nathanson DA, Gini B, Mottahedeh J, Visnyei K, Koga T, Gomez G, Eskin A, Hwang K, Wang J, Masui K (2014). Targeted therapy resistance mediated by dynamic regulation of extrachromosomal mutant EGFR DNA. Science.

[18] Siegel JJ, Amon A (2012). New insights into the troubles of aneuploidy. Annu Rev Cell Dev Biol.

[19] Hiley CT, Swanton C (2014). Spatial and temporal cancer evolution: causes and consequences of tumour diversity. Clin Med (Lond).

[20] Venkitaraman AR (2014). Cancer suppression by the chromosome custodians, BRCA1 and BRCA2. Science.

[21] Lord CJ, Ashworth A (2016). BRCAness revisited. Nat Rev Cancer.

[22] Roschke AV, Kirsch IR (2005). Targeting cancer cells by exploiting karyotypic complexity and chromosomal instability. Cell Cycle.

[23] Carter SL, Eklund AC, Kohane IS, Harris LN, Szallasi Z (2006). A signature of chromosomal instability inferred from gene expression profiles predicts clinical outcome in multiple human cancers. Nat Genet.

[24] Tam AS, Sihota TS, Milbury KL, Zhang A, Mathew V, Stirling PC (2019). Selective defects in gene expression control genome instability in yeast splicing mutants. Mol Biol Cell.

[25] Habermann JK, Doering J, Hautaniemi S, Roblick UJ, Bundgen NK, Nicorici D, Kronenwett U, Rathnagiriswaran S, Mettu RK, Ma Y (2009). The gene expression signature of genomic instability in breast cancer is an independent predictor of clinical outcome. Int J Cancer.

[26] Wang T, Wang G, Zhang X, Wu D, Yang L, Wang G, Hao D (2017). The expression of miRNAs is associated with tumour genome instability and predicts the outcome of ovarian cancer patients treated with platinum agents. Sci Rep.

[27] Zeng X, Liu L, Lu L, Zou Q (2018). Prediction of potential disease-associated microRNAs using structural perturbation method. Bioinformatics.

[28] Zhang X, Zou Q, Rodriguez-Paton A, Zeng X (2019). Meta-Path Methods for Prioritizing Candidate Disease miRNAs. IEEE/ACM Trans Comput Biol Bioinform.

[29] Ulitsky I, Bartel DP (2013). lincRNAs: genomics, evolution, and mechanisms. Cell.

[30] Mercer TR, Dinger ME, Mattick JS (2009). Long non-coding RNAs: insights into functions. Nat Rev Genet.

[31] Kretz M, Siprashvili Z, Chu C, Webster DE, Zehnder A, Qu K, Lee CS, Flockhart RJ, Groff AF, Chow J (2013). Control of somatic tissue differentiation by the long non-coding RNA TINCR. Nature.

[32] Liu H (2016). Linking lncRNA to genomic stability. Sci China Life Sci.

[33] Munschauer M, Nguyen CT, Sirokman K, Hartigan CR, Hogstrom L, Engreitz JM, Ulirsch JC, Fulco CP, Subramanian V, Chen J (2018). The NORAD lncRNA assembles a topoisomerase complex critical for genome stability. Nature.

[34] Betts JA, Moradi Marjaneh M, Al-Ejeh F, Lim YC, Shi W, Sivakumaran H, Tropee R, Patch AM, Clark MB, Bartonicek N (2017). Long Noncoding RNAs CUPID1 and CUPID2 Mediate Breast Cancer Risk at 11q13 by Modulating the Response to DNA Damage. Am J Hum Genet.

[35] Polo SE, Blackford AN, Chapman JR, Baskcomb L, Gravel S, Rusch A, Thomas A, Blundred R, Smith P, Kzhyshkowska J (2012). Regulation of DNA-end resection by hnRNPU-like proteins promotes DNA double-strand break signaling and repair. Mol Cell.

[36] Sharma V, Khurana S, Kubben N, Abdelmohsen K, Oberdoerffer P, Gorospe M, Misteli T (2015). A BRCA1-interacting lncRNA regulates homologous recombination. EMBO Rep.

[37] Bao S, Zhao H, Yuan J, Fan D, Zhang Z, Su J, Zhou M (2020). Computational identification of mutator-derived lncRNA signatures of genome instability for improving the clinical outcome of cancers: a case study in breast cancer. Brief Bioinform.

[38] van Rhijn BWG, Mertens LS, Mayr R, Bostrom PJ, Real FX, Zwarthoff EC, Boormans JL, Abas C, van Leenders G, Gotz S (2020). FGFR3 mutation status and FGFR3 expression in a large bladder cancer cohort treated by radical cystectomy: implications for anti-FGFR3 treatment? (dagger). Eur Urol.

[39] Kronenwett U, Ploner A, Zetterberg A, Bergh J, Hall P, Auer G, Pawitan Y (2006). Genomic instability and prognosis in breast carcinomas. Cancer Epidemiol Biomarkers Prev.

[40] Mettu RK, Wan YW, Habermann JK, Ried T, Guo NL (2010). A 12-gene genomic instability signature predicts clinical outcomes in multiple cancer types. Int J Biol Markers.

[41] Tubbs A, Nussenzweig A (2017). Endogenous DNA Damage as a Source of Genomic Instability in Cancer. Cell.

[42] Bhan A, Soleimani M, Mandal SS (2017). Long noncoding RNA and cancer: a new paradigm. Cancer Res.

[43] Wang L, Cho KB, Li Y, Tao G, Xie Z, Guo B (2019). Long Noncoding RNA (lncRNA)-Mediated Competing Endogenous RNA networks provide novel potential biomarkers and therapeutic targets for colorectal cancer. Int J Mol Sci.

[44] Zhuang C, Ma Q, Zhuang C, Ye J, Zhang F, Gui Y (2019). LncRNA GClnc1 promotes proliferation and invasion of bladder cancer through activation of MYC. FASEB J.

[45] Munschauer M, Nguyen CT, Sirokman K, Hartigan CR, Hogstrom L, Engreitz JM, Ulirsch JC, Fulco CP, Subramanian V, Chen J (2018). Publisher Correction: The NORAD lncRNA assembles a topoisomerase complex critical for genome stability. Nature.

[46] Hu WL, Jin L, Xu A, Wang YF, Thorne RF, Zhang XD, Wu M (2018). GUARDIN is a p53-responsive long non-coding RNA that is essential for genomic stability. Nat Cell Biol.

[47] Fowler AK, Hewetson A, Agrawal RG, Dagda M, Dagda R, Moaddel R, Balbo S, Sanghvi M, Chen Y, Hogue RJ (2012). Alcohol-induced one-carbon metabolism impairment promotes dysfunction of DNA base excision repair in adult brain. J Biol Chem.

[48] Kruman II, Henderson GI, Bergeson SE (2012). DNA damage and neurotoxicity of chronic alcohol abuse. Exp Biol Med (Maywood).

[49] Singh N, Traisak P, Martin KA, Kaplan MJ, Cohen PL, Denny MF (2014). Genomic alterations in abnormal neutrophils isolated from adult patients with systemic lupus erythematosus. Arthritis Res Ther.

[50] Souliotis VL, Vlachogiannis NI, Pappa M, Argyriou A, Ntouros PA, Sfikakis PP (2019). DNA damage response and oxidative stress in systemic autoimmunity. Int J Mol Sci.

[51] Zhang G, Chen X, Ma L, Ding R, Zhao L, Ma F, Deng X (2020). LINC01419 facilitates hepatocellular carcinoma growth and metastasis through targeting EZH2-regulated RECK. Aging (Albany NY).

[52] Wang LL, Zhang L, Cui XF (2019). Downregulation of long noncoding RNA LINC01419 inhibits cell migration, invasion, and tumor growth and promotes autophagy via inactivation of the PI3K/Akt1/mTOR pathway in gastric cancer. Ther Adv Med Oncol.

[53] Gu Z, Wu S, Wang J, Zhao S (2020). Long non-coding RNA LINC01419 mediates miR-519a-3p/PDRG1 axis to promote cell progression in osteosarcoma. Cancer Cell Int.

[54] Xie Q, Chen S, Tian R, Huang X, Deng R, Xue B, Qin Y, Xu Y, Wang J, Guo M (2018). Long Noncoding RNA ITPRIP-1 positively regulates the innate immune response through promotion of oligomerization and activation of MDA5. J Virol.

[55] Zhang X, Zhang J, Zhao W, Dong X, Xin P, Liu X, Li X, Jing Z, Zhang Z, Kong C et al: Long non-coding RNA LINC02446 suppresses the proliferation and metastasis of bladder cancer cells by binding with EIF3G and regulating the mTOR signalling pathway. Cancer Gene Ther 2021. 10.1038/s41417-020-00285-2 33526846

[56] Ahmad F, Mahal V, Verma G, Bhatia S, Das BR (2018). Molecular investigation of FGFR3 gene mutation and its correlation with clinicopathological findings in Indian bladder cancer patients. Cancer Rep (Hoboken).

[57] Borkowska EM, Traczyk-Borszynska M, Kutwin P, Pietrusinski M, Jablonowski Z, Borowiec M (2019). Usefulness of droplet digital PCR and Sanger sequencing for detection of FGFR3 mutation in bladder cancer. Urol Oncol.

