# Supplementary material for: Calculated identification of mutator-derived lncRNA signatures of genomic instability to predict the clinical outcome of muscle-invasive bladder cancer
Source: Cancer Cell Int. 2021 Sep 8;21:476. doi: 10.1186/s12935-021-02185-3 (PMC8424867; doi:10.1186/s12935-021-02185-3)
Supplement: Supplementary file 1 — Additional file 1: Fig. S1. (a) Heatmap of expression of 43 differential lncRNAs. (b) ROC curve and AUC based on the testing cohort at 5 years. (c) ROC curve and AUC based on the entire TCGA MIBC cohort at 5 years. [file 12935_2021_2185_MOESM1_ESM.docx]

**
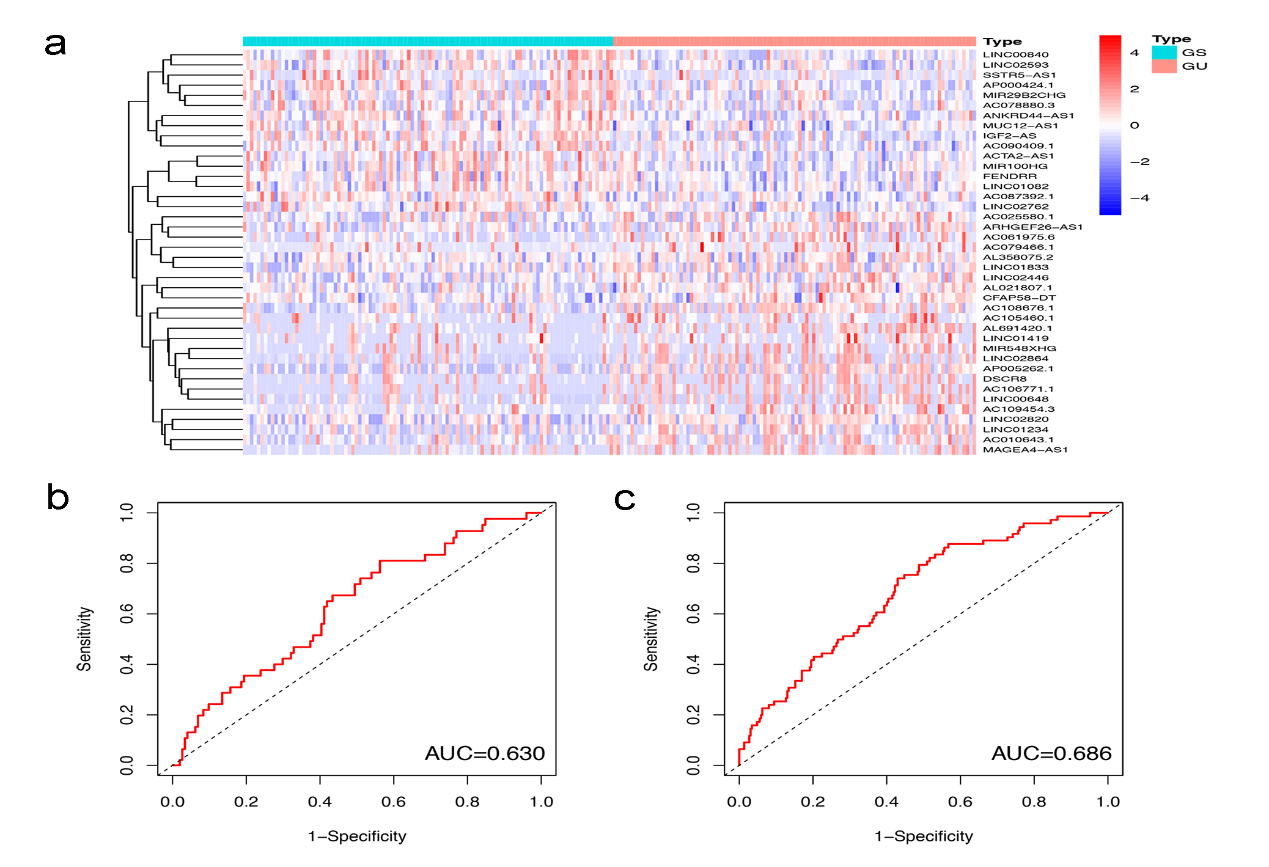
**

**Additional file 1: Fig. S1** (a)Heatmap of expression of 43 differential lncRNAs. (b) ROC curve and AUC based on the testing cohort at 5 years. (c) ROC curve and AUC based on the entire TCGA MIBC cohort at 5 years.
